# Non-Sterilized Fermentative Production of Polymer-Grade L-Lactic Acid by a Newly Isolated Thermophilic Strain *Bacillus* sp. 2–6

**DOI:** 10.1371/journal.pone.0004359

**Published:** 2009-02-04

**Authors:** Jiayang Qin, Bo Zhao, Xiuwen Wang, Limin Wang, Bo Yu, Yanhe Ma, Cuiqing Ma, Hongzhi Tang, Jibin Sun, Ping Xu

**Affiliations:** 1 Tianjin Industrial Biotechnology R&D Center, Chinese Academy of Sciences, Tianjin, People's Republic of China; 2 Institute of Microbiology, Chinese Academy of Sciences, Beijing, People's Republic of China; 3 State Key Laboratory of Microbial Technology, Shandong University, Jinan, People's Republic of China; 4 Key Laboratory of Microbial Metabolism, Ministry of Education, School of Life Sciences and Biotechnology, Shanghai Jiao Tong University, Shanghai, People's Republic of China; Center for Genomic Regulation, Spain

## Abstract

**Background:**

The demand for lactic acid has been increasing considerably because of its use as a monomer for the synthesis of polylactic acid (PLA), which is a promising and environment-friendly alternative to plastics derived from petrochemicals. Optically pure l-lactic acid is essential for polymerization of PLA. The high fermentation cost of l-lactic acid is another limitation for PLA polymers to compete with conventional plastics.

**Methodology/Principal Findings:**

A *Bacillu*s sp. strain 2–6 for production of l-lactic acid was isolated at 55°C from soil samples. Its thermophilic characteristic made it a good lactic acid producer because optically pure l-lactic acid could be produced by this strain under open condition without sterilization. In 5-liter batch fermentation of *Bacillus* sp. 2–6, 118.0 g/liter of l-lactic acid with an optical purity of 99.4% was obtained from 121.3 g/liter of glucose. The yield was 97.3% and the average productivity was 4.37 g/liter/h. The maximum l-lactic acid concentration of 182.0 g/liter was obtained from 30-liter fed-batch fermentation with an average productivity of 3.03 g/liter/h and product optical purity of 99.4%.

**Conclusions/Significance:**

With the newly isolated *Bacillu*s sp. strain 2–6, high concentration of optically pure l-lactic acid could be produced efficiently in open fermentation without sterilization, which would lead to a new cost-effective method for polymer-grade l-lactic acid production from renewable resources.

## Introduction

Lactic acid, also named 2-hydroxypropanoic acid, is a versatile chemical widely used in food, cosmetic, pharmaceutical, textile and chemical industries. Recently, its application has been extended to the plastics industry, where polylactic acid (PLA) is utilized as a biodegradable and biocompatible plastic material [1]–[6]. The environment-friendly characteristic of PLA, which may reduce the net emission of carbon dioxide and the demand for petroleum, results in wide uses and increased market values of PLA and its monomers [2], [3]. Lactic acid exists in two optically active isomeric forms, l(+) and d(−). Because the physical properties and stability of PLA depend on the isomeric composition of lactic acid, the optically pure lactic acid is essential for polymerization [7]–[9].

Lactic acid can be produced by either chemical synthesis or fermentation. Chemical synthesis provides only the racemic lactic acid, whereas, fermentation technology can produce single desired stereoisomer (l(+) or d(−) only) or a racemic mixture (dl) of lactic acid using different organisms [3], [4], [10]. High product specificity, low costs of substrates, low production temperature and low energy consumption are all advantages of the biotechnological methods for lactic acid production over the chemical methods [3], [5].

Currently, sterilization is necessary for fermentative production of l-lactic acid. *Lactobacillus* species and *Rhizopus oryzae*, which have optimal fermentation temperature of 30–42°C, are usually used for industrial applications [3], [6]. To the best of our knowledge, d- and dl-lactic acid producers which are widespread in the earth have similar optimal fermentation temperatures. Therefore, it's hard to avoid contaminations if the medium were not sterilized.

Recently, some thermophilic *Bacillus* species were suggested to be new lactic acid producers because of their higher fermentation temperature [11]. Michelson et al. [12] reported lactic acid production using *B. coagulans* SIM-7 DSM 14043 and 91.6 g/liter of lactic acid was obtained in fed-batch fermentation. Rosenberg et al. [13] reported lactic acid production using immobilized cells of *B. coagulans* CCM 4318 and 77.5 g/liter of lactic acid could be produced. Other thermophilic *Bacillus* species used for lactic acid production include *Bacillus* sp. 17C5 and *B. coagulans* TB/04, but both of them can produce only 55 g/liter of lactic acid [11], [14]. Thus the limitation of using thermophilic *Bacillus* species is likely due to their relatively low lactic acid productivities.

In this paper, we report non-sterilized fermentative production of polymer-grade l-lactic acid by a newly isolated thermophilic strain *Bacillus* sp. 2–6. Fermentation conditions were optimized. High yield, productivity and optical purity of l-lactic acid were obtained in batch and fed-batch open fermentations. These results indicate that *Bacillus* sp. 2–6 is a promising new l-lactic acid producer.

## Results

### Isolation of bacteria for lactic acid production at 55°C

Strain 2–6 was selected as the best producer of l-lactic acid from the initial 730 strains isolated from 7 soil samples. It was tentatively identified as *Bacillus* species according to its 16S rRNA gene sequence (GenBank accession number: EU307106). The enantiomeric excess (ee) value of l-lactic acid produced by this strain was compared with those by some *Lactobacillus* species under same conditions (Table S1). Strain 2–6 showed an ee value of 97.3%, much higher than that of l-lactic acid produced by these *Lactobacillus* species, among which the best result obtained was only 84.4% with *L. pentosus* DSM 20314.

### NAD-dependent lactate dehydrogenase activity in *Bacillus* sp. 2–6

**Figure 1:**
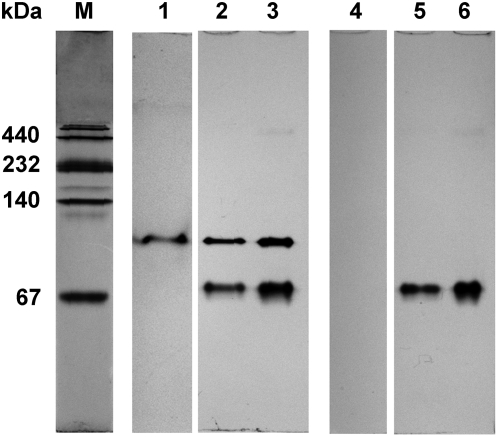
Active staining of NAD-dependent lactate dehydrogenases after native polyacrylamide gel electrophoresis (PAGE). Cell extracts of *Bacillus* sp. 2–6 (lane 1 and 4), *L. pentosus* DSM 20314 (lane 2 and 5) and *L. plantarum* DSM 20205 (lane 3 and 6) were used for the native PAGE. dl-Lactate was used as substrate for active staining in lane 1, 2 and 3, while D-lactate was used as substrate in lane 4, 5 and 6.

In order to investigate why the l-lactic acid produced by *Bacillus* sp. 2–6 had better optical purity, the cell extract was used to test if there was d-lactate dehydrogenase activity in the strain. *L. pentosus* DSM 20314 and *L. plantarum* DSM 20205, which showed the best and the worst ee values respectively among these *Lactobacillus* strains in Table S1, were selected as controls. No NAD-dependent d-lactate dehydrogenase activity was detected in strain 2–6, while the specific activities in *L. pentosus* DSM 20314 and *L. plantarum* DSM 20205 were 0.90±0.01 U/mg and 1.49±0.05 U/mg, respectively.

d-Lactate dehydrogenase activities were detected in *L. pentosus* DSM 20314 and *L. plantarum* DSM 20205 but not in *Bacillus* sp. 2–6 by active staining after native polyacrylamide gel electrophoresis (PAGE), while activities of l-lactate dehydrogenase were detected in all the three strains (Figure 1).

### Selection of optimal fermentation temperature and initial glucose concentration

The l-lactic acid productions were 60.5 g/liter and 59.0 g/liter at 45°C and 55°C, respectively. Only 41.0 g/liter l-lactic acid was produced at 60°C under the same conditions. The best l-lactic acid concentration of 66.0 g/liter was obtained at 50°C and this temperature was thus selected for further investigations.

**Figure 2:**
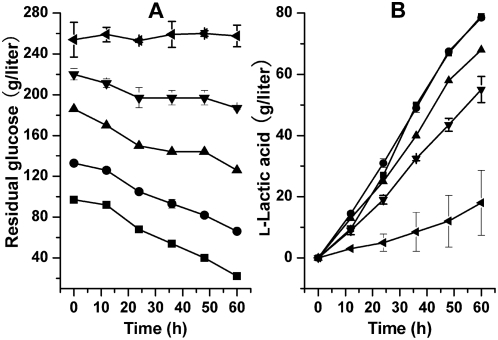
Effects of different initial glucose concentrations on l-lactic acid production by *Bacillus* sp. 2–6. (A) Time courses of sugar consumption. (B) Time courses of l-lactic acid production. The initial glucose concentrations used were at 97 g/liter (▪), 133 g/liter (•), 186 g/liter (▴), 220 g/liter (▾) and 254 g/liter (◂).

To investigate the substrate tolerance of *Bacillus* sp. 2–6, different initial glucose concentrations were used for l-lactic acid production (Figure 2). With 254 g/liter of initial glucose, no glucose was consumed (Figure 2A) and l-lactic acid was hardly produced (Figure 2B). When the initial glucose concentration was 220 g/liter or 186 g/liter, l-lactic acid production was limited and the titer was 55 g/liter or 68 g/liter at 60 h which was 69.6% or 86.1% of that with 97 g/liter of initial glucose. No limitation was observed with 133 g/liter of initial glucose because it resulted in similar l-lactic acid production to that with 97 g/liter of initial glucose. Therefore, initial glucose concentration between 97 g/liter and 133 g/liter was selected for further investigations.

### Statistical optimization of fermentation medium

Statistical approaches were used in this study for optimization of fermentation medium (Supplementary Information S1). The optimization began with the selection of the right nitrogen sources. Soy peptide (Lenon Bio-Tech, Ltd, China), yeast extract (YE) (Angel Yeast Co., Ltd, China) and cottonseed protein (Aoboxing Universeen Bio-Tech Co., Ltd, China) were selected from six organic nitrogen sources as most suitable for *Bacillus* sp. 2–6 (data not shown). Effects of several inorganic nitrogen sources and trace elements on l-lactic acid production were investigated and the results showed that NaNO_3_, NH_4_Cl and Mg^2+^ had positive effect on l-lactic acid production when YE (5 g/liter) was used as the sole nitrogen source (Table 1). Many studies revealed that vitamins were required during lactic acid production by thermophilic *Bacillus* [15]–[17]. Therefore, a vitamin solution, along with soy peptide, YE, cottonseed protein and inorganic nitrogen sources, were used to compose the Plackett-Burman design (Tables 2 and 3). The results showed that soy peptide (*X*_1_) and YE (*X*_2_) had significant positive influence on l-lactic acid production [18]. In the steepest ascent experiment, the concentration of l-lactic acid reached its maximum of 115.0 g/liter at *X*_1_ = 7 g/liter, *X*_2_ = 12 g/liter. Central composite design was carried out around this point and the maximum value of l-lactic acid (116.7 g/liter) was attained at soy peptide and YE concentrations of 5.1 g/liter (*x*_1_ = −0.4680) and 14.3 g/liter (*x*_2_ = −0.3768), respectively (Figure S1).

**Table 1:** Effects of inorganic nitrogen sources and trace elements on l-lactic acid production.

Compound added	l-Lactic acid (g/liter)
None	23.8±0.4 a
(NH_4_)_2_SO_4_ (1 g/liter)	22.0±0.0
(NH_4_)_2_HPO_4_ (1 g/liter)	19.0±0.0
NH_4_NO_3_ (1 g/liter)	24.0±0.7
NaNO_3_ (1 g/liter)	33.0±1.4
NH_4_Cl (1 g/liter)	27.0±0.0
CO(NH_2_)_2_ (1 g/liter)	26.3±0.4
Ammonium citrate (1 g/liter)	21.5±0.0
MgSO_4_ (1 g/liter)	29.3±0.4
MnSO_4_ (0.3 g/liter)	21.0±0.7

a Values in Table 1 and Table 3 are the average±standard deviation of three repeated fermentations.

**Table 2:** Nutrient supplements screening by Plackett-Burman design.

Factor		Level	
Variable	Nutrient	Low (−1)	High (+1)
*X*_1_	Soy peptide (g/liter)	1	3
*X*_2_	YE (g/liter)	1	3
*X*_3_	Cottonseed protein (g/liter)	1	3
*X*_4_	NaNO_3_ (g/liter)	0	1
*X*_5_	NH_4_Cl (g/liter)	0	1
*X*_6_	MgCl_2_ (g/liter)	0	0.1
*X*_7_	Vitamin solution a (ml/liter)	0	2

a Vitamin solution: biotin 20 mg/liter, thiamine 500 mg/liter, niacin 500 mg/liter, folic acid 30 mg/liter.

**Table 3:** Design and results of the Plackett-Burman design.

Run	Coded variable a level							Response (*Y* (g/liter))
	*x*_1_	*x*_2_	*x*_3_	*x*_4_	*x*_5_	*x*_6_	*x*_7_	
1	+1	−1	+1	−1	−1	−1	+1	26.8±0.4
2	+1	+1	−1	+1	−1	−1	−1	44.5±0.0
3	−1	+1	+1	−1	+1	−1	−1	40.5±1.4
4	+1	−1	+1	+1	−1	+1	−1	27.5±0.0
5	+1	+1	−1	+1	+1	−1	+1	45.0±0.7
6	+1	+1	+1	−1	+1	+1	−1	46.5±0.7
7	−1	+1	+1	+1	−1	+1	+1	39.0±0.0
8	−1	−1	+1	+1	+1	−1	+1	20.5±0.7
9	−1	−1	−1	+1	+1	+1	−1	18.5±1.4
10	+1	−1	−1	−1	+1	+1	+1	26.8±1.1
11	−1	+1	−1	−1	−1	+1	+1	29.5±0.0
12	−1	−1	−1	−1	−1	−1	−1	15.3±1.8

a *x*_1_, *x*_2_, *x*_3_, *x*_4_, *x*_5_, *x*_6_, *x*_7_ are the coded levels of soy peptide (*X*_1_), YE (*X*_2_), cottonseed protein (*X*_3_), NaNO_3_ (*X*_4_), NH_4_Cl (*X*_5_), MgCl_2_ (*X*_6_) and vitamin solution (*X*_7_) in Table 2, respectively.

The dosage of soy peptide and YE were further optimized by testing the cost-effectiveness of the medium. Soy peptide (*X*_1_) and YE (*X*_2_) concentrations at 1.2 g/liter and 12.6 g/liter resulted in 110.9 g/liter of l-lactic acid production, amounting to 95.0% of the theoretical maximum yield (Figure S2). This medium saved 3.9 g/liter of soy peptide and 1.7 g/liter of YE, and represented the most cost-effective medium.

According to the results of the statistically designed experiments and the investigation on initial glucose concentration, a cost-effective medium for l-lactic acid production by *Bacillus* sp. 2–6 was obtained (g/liter): glucose 97–133, YE 12.6, soy peptide 1.2, cottonseed protein 3, NaNO_3_ 1, NH_4_Cl 1.

### Batch and fed-batch fermentations

**Figure 3:**
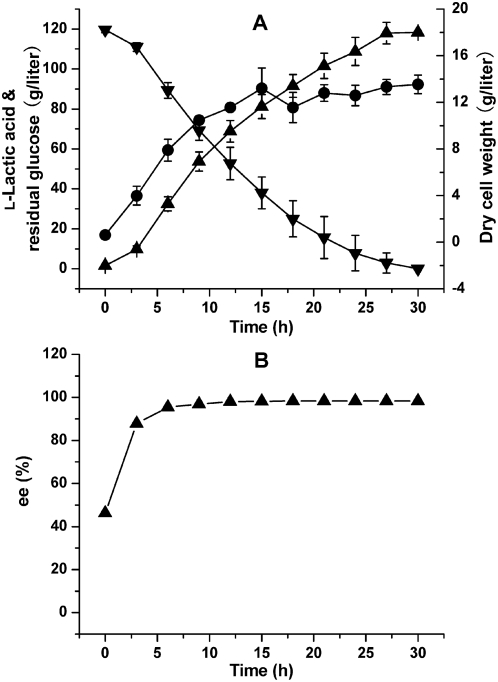
Batch fermentations of l-lactic acid by *Bacillus* sp. 2–6 without sterilization. (A) Time courses of sugar consumption and l-lactic acid production. (B) Time courses of enantiomeric excess (ee) values during the fermentation. Symbols represent: ▾, residual glucose; ▴, l-lactic acid; ▸, ee values of l-lactic acid; •, dry cell weight.

Batch fermentations were performed in a 5-liter bioreactor using the optimal medium and strain *Bacillus* sp. 2–6. Fermentation profiles suggested two distinct phases due to decoupling of growth from lactic acid production around 15 h at which the dry cell weight reached the maximum of 13.2 g/liter (Figure 3A). The average l-lactic acid productivities of the two phases (0–15 h, 15–27 h) were 5.41 g/liter/h and 3.07 g/liter/h, respectively. The formation of l-lactic acid finished at 27 h when the residual glucose was completely consumed, and the l-lactic acid titer climbed to its maximum at 118.0 g/liter. The yield was 97.3% of the theoretical value and no other organic acids could be detected. The average productivity from 0 to 27 h was 4.37 g/liter/h. At 0 h, the ee value of l-lactic acid was 62.1%, indicating that there was 0.35 g/liter d-lactic acid mainly from the medium. The ee value dramatically increased to 88.9% after 3 h and then reached 99.4% at 18 h (Figure 3B).

**Figure 4:**
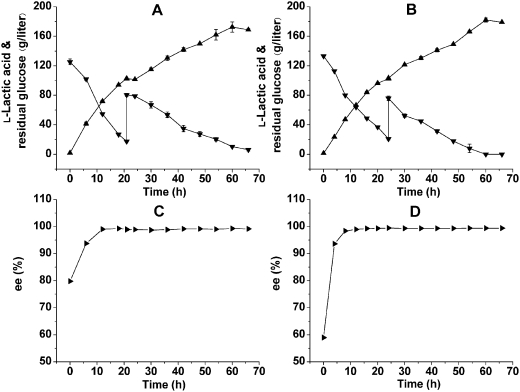
Fed-batch fermentations of l-lactic acid by *Bacillus* sp. 2–6 under non-sterilized conditions. (A) Time courses of sugar consumption and l-lactic acid production in 5-liter fed batch. (B) Time courses of ee values during 5-liter fed-batch fermentation. (C) Time courses of sugar consumption and l-lactic acid production in 30-liter fed batch. (D) Time courses of ee values during 30-liter fed-batch fermentation. Symbols represent: ▾, residual glucose; ▴, l-lactic acid; ▸, ee values of l-lactic acid.

Fed-batch fermentations were firstly tested with three different feeding strategies: constant glucose-concentration feeding strategy, exponential feeding strategy and pulse feeding strategy. It was shown that the first and the third strategy gave similar results and both of them were much better than the second strategy after 48 h fermentation (Table 4). Considering that the pulse feeding strategy was easier to operate and therefore may be more suitable for industrial scale production, it was used in further investigations. Results of 5-liter fed-batch fermentations are shown in Figure 4. The feeding started at 21 h when the residual glucose concentration was below 20 g/liter. The curve of l-lactic acid production can be divided into two parts (Figure 4A). Before 21 h, 102.5 g/liter of l-lactic acid was produced and the average productivity reached 4.88 g/liter/h. From 21 h to 60 h, the concentration of l-lactic acid climbed from 102.5 g/liter to 172.5 g/liter and the average productivity was only 1.79 g/liter/h. At the end of the fed-batch fermentation, 178.2 g/liter of glucose was consumed and the final l-lactic acid concentration was 172.5 g/liter. The yield was 95.8% of the theoretical value and the average productivity was 2.88 g/liter/h. The optical purity of l-lactic acid during the fermentation is shown in Figure 4C. The ee value of l-lactic acid increased from 79.8% at 0 h to 93.7% at 6 h, and the final ee value at 60 h was 99.2%. Similar results were obtained in 30-liter fed-batch fermentation (Figure 4B and 4D). The final concentration, average productivity and ee value of l-lactic acid was 182.0 g/liter, 3.03 g/liter/h and 99.4%, respectively.

**Table 4:** l-Lactic acid production with different feeding strategies.

Feeding strategy	l-Lactic acid (g/liter)	Productivity (g/liter/h)
Pulse feeding strategy	150.0	3.2
Constant glucose-concentration feeding strategy	144.0	3.0
Exponential feeding strategy (*μ* = 0.51/h)	122.0	2.5
Exponential feeding strategy (*μ* = 0.15/h)	140.5	2.9

## Discussion

To date, microorganisms including *Lactobacillus* species, *Lactococcus* species, *Streptococcus* species, *Enterococcus* species, *Sporolactobacillus* species and fungi have been reported to be lactic acid producers. *Lactobacillus* species have demonstrated their ability to produce high yield of lactic acid [19], [20]. However, a fatal limitation is that the optical purity of lactic acid they produce may not satisfy the demands of polymeric grade PLA production. Efforts have been made to inactivate the lactate dehydrogenase responsible for d- or l-lactic acid formation [21]–[23], however, the antibiotic resistant markers in the engineered strains would compromise their applications in industry [24]. Fungal fermentation requires vigorous aeration because they are obligate aerobe. Additionally, low reaction rate (below 3 g/liter/h) caused by mass transfer limitation and by-products (fumaric acid and ethanol, etc.) accumulation were observed in fungal fermentation [25], [26]. Other lactic acid producers, such as *Lactococcus* species, *Streptococcus* species and *Enterococcus* species are in limited usage due to their low productivities.

Moreover, all of these strains have an optimal fermentation temperature of 30–42°C, which makes it difficult to avoid the risk of contaminations caused by dl-lactic acid producers such as *L. plantarum*, therefore, compromises the enantiomeric purity of the lactic acid. Compared with these lactic acid producers, strain *Bacillus* sp. 2–6 has a much higher fermentation temperature at about 50°C, which enables non-sterilized batch and fed-batch fermentations for l-lactic acid production. In this study, more than twenty batches were performed and no contamination occurred during open operations. The easy-to-handle strain also provided an opportunity to avoid the degradation of substrate sugars and other nutritional elements for lactic acid fermentations during the sterilization. The Maillard reaction, which leads to production of unfavorable furfural compounds and subsequently increases the colourity of the fermentation broth, was also avoided [27], [28]. Practically, non-sterilization means lower equipment requirement and energy consumption, the omission of the sterilization equipments and the decrease of labor cost. Non-sterilization and open fermentation would also effectively decrease the fermentation cost in raw material, decoloration and other related operations, which is especially important for the production of the low-value high-volume chemical l-lactic acid to compete with traditional options [3].

d-Lactate and l-lactate are formed from pyruvate by reductive reactions with stereospecific NAD-dependent lactate dehydrogenases. Many lactic acid bacteria possess NAD-dependent d-lactate dehydrogenase, including l-lactate producers such as *L. casei* and *L. pentosus*, and dl-lactate producers such as *L. plantarum* [10], [22], [23], [29]. It is considered that no *Lactobacillus* forms only l-lactate [30]. *Streptococci*, on the other hand, make only l-lactate and have no d-lactate dehydrogenase [30]. Inactivation of d-lactate dehydrogenase successfully improved the optical purity of l-lactic acid in *L. casei* [23]. In this study, *Bacillus* sp. 2–6 produced optically purer l-lactic acid than some *Lactobacillus* species under same conditions (Table S1). In batch and fed-batch fermentations, the final ee values of l-lactic acid produced by strain 2–6 were at about 99.3%. In fact, these values were no less than 99.5% considering the contribution of medium components to the content of d-lactic acid. The high optical purity may be due to the undetectable activity or lack of an NAD-dependent d-lactate dehydrogenase in *Bacillus* sp. 2–6.

In this work, the average productivities for batch and fed-batch fermentations were 4.37 g/liter/h and 3.03 g/liter/h, respectively. To our knowledge, two representative l-lactic acid producers of *Lactobacillus* species, *L. casei* LA-04-1 and *L. lactis* BME5-18M, had average productivities of 2.14 g/liter/h and 2.2 g/liter/h, respectively [19], [20], [31]. Compared with *Lactobacillus* species, the higher productivity of l-lactic acid by strain 2–6 was likely due to the higher cell mass. During batch fermentation, the dry cell weight of *Bacillus* sp. 2–6 reached 13.2 g/liter, much higher than that obtained from *L. casei* LA-04-1 and *L. lactis* BME5-18M, which was 4.3 g/liter and 2.7 g/liter, respectively [19], [20]. The byproducts of the batch fermentation by *Bacillus* sp. 2–6 were determined using an HPLC equipped with an organic acid column. No obvious other organic acid was detected indicating that this strain was homofermentative and most of the glucose was converted to lactic acid.

In conclusion, *Bacillus* sp. 2–6 has merits of high fermentation temperature, high productivity, high yield and high product optical purity. Its thermophilic characteristic made it a good lactic acid producer because open fermentations without sterilization are favorable in reducing the cost of l-lactic acid production. Combined with the excellent production traits, *Bacillus* sp. 2–6 will be more suitable in industrial production of polymer-grade l-lactic acid.

## Materials and Methods

### Isolation of bacteria for lactic acid production

Soil samples were collected from various areas, including farmland, gardens and lands near milk factories. Approximately 2 g of each was enriched in 50 ml of nutrient liquid medium and incubated at 55°C without agitation for 6 h. An aliquot of the broth was plated on nutrient agar medium containing (g/liter): glucose 50, YE 10, CaCO_3_ 20, agar 20. After 24 h of incubation at 55°C, representative colonies were selected based on colony size and acid production zone. Then the selected colonies were incubated in medium containing (g/liter): glucose 150, YE 20, CaCO_3_ 75. After 48 h of incubation at 55°C without agitation, the strain that produced the most l-lactic acid, designated as 2–6, was selected for further analysis.

### Microorganisms and culture conditions

Strain 2–6, *L. casei* DSM 20011, *L. plantarum* DSM 20205, *L. pentosus* DSM 20314 and *Lactobacillus* sp. DSM 20605 were studied in this work. These strains were maintained on MRS agar slants containing (g/liter): peptone 10, ‘Lab-Lemco’ powder 8, YE 4, glucose 20, triammonium citrate 2, sodium acetate 5, K_2_HPO_4_ 2, MgSO_4_·7H_2_O 0.2, MnSO_4_·H_2_O 0.05, CaCO_3_ 10. The pH was adjusted to 6.5. The slants were incubated at 50°C or 37°C and the fully grown slants were stored at 4°C.

The medium for inoculation contained (g/liter): glucose 70, soybean peptone 5, YE 10, CaCO_3_ 20 (GSY medium). The seed culture was prepared as follows: a loop of cells from the fully-grown slant was inoculated into 50 ml of the above sterile medium in 300-ml Erlenmeyer flasks and incubated for 12 h at 50°C without agitation. Then the seed culture was inoculated into Erlenmeyer flasks or bioreactors (inoculum volume at 10%) for lactic acid production.

### Selection of optimal fermentation temperature and initial glucose concentration

The optimal fermentation temperature of *Bacillus* sp. 2–6 was tested using the medium contained (g/liter): glucose 100, YE 20, CaCO_3_ 60. Fermentations were conducted at 45°C, 50°C, 55°C and 60°C, respectively, and samples were taken after 48 h of fermentation.

For studying initial glucose concentration, the following medium was used (g/liter): YE 20, CaCO_3_ 70. The initial glucose concentrations studied were at 97 g/liter, 133 g/liter, 186 g/liter, 220 g/liter and 254 g/liter. Samples were taken every 12 h and the concentrations of residual glucose and l-lactic acid were determined. All the fermentations mentioned above were conducted at 50°C in 300-ml Erlenmeyer flasks containing 100 ml media without agitation.

### Assay of NAD-dependent D-lactate dehydrogenase

To assay the enzymatic activities in *Bacillus* sp. 2–6, *L. pentosus* DSM 20314 and *L. plantarum* DSM 20205, cells grown in GSY medium were harvested during the exponential phase. Then they were washed and resuspended in 0.1 M potassium phosphate buffer (pH 7.4) and disrupted by sonication in an ice bath. The disrupted cells were centrifuged for 15 min at 10,000×*g*, and the supernatant was used as the crude cell extract. d-Lactate dehydrogenase (EC 1.1.1.28) was assayed photometrically (340 nm) by reactions coupled to NAD reduction as described [29]. The temperatures for the assays were 37°C and 50°C for *Bacillus* sp. 2–6 and 37°C for *Lactobacillus* strains. One unit (1 U) was defined as the amount of enzyme accumulating 1 µmol of NADH per minute. Protein concentrations were determined as described previously [32], with bovine serum albumin as standard.

Active staining of NAD-dependent lactate dehydrogenases after native PAGE was performed according to the previous report [33] with some modifications. Native PAGE was performed on a 9% native polyacrylamide gel. After electrophoresis, gels were cut into five parts. One was fixed with 12.5% trichloroacetic acid and stained with Coomassie Brilliant Blue R-250. The other four were soaked in the substrate solution (100 mM Tris–HCl buffer, pH 8.0, containing 0.1 mM phenazine methosulfate, 0.1 mM nitrotetrazolium blue chloride, 10 mM NAD and 100 mM d-lactate or dl-lactate) separately with gentle shaking for 60 min. Cell extracts of *Bacillus* sp. 2–6, *L. pentosus* DSM 20314 and *L. plantarum* DSM 20205 were concentrated and used for the native PAGE.

### Statistical optimization of fermentation medium

Statistical approaches including Plackett-Burman design, steepest ascent design and central composite design were used in this study [34], [35]. SAS package (version 9.0, SAS Institute Inc., USA) was used for all the statistical analysis and the response surface plotting. Besides the studied components, the fermentation media for all the statistical optimization experiments contained 150 g/liter of glucose and 80 g/liter of CaCO_3_. All of the experiments in this part were conducted in 300-ml Erlenmeyer flasks containing 100 ml of each medium with shaking at 100 rpm on a rotary shaker at 50°C. Samples were taken after 24 h and the concentrations of l-lactic acid were determined.

### Batch and fed-batch fermentations

Batch fermentations of strain 2–6 were carried out in a 5-liter bioreactor (BIOSTAT B, B. Braun Biotech International GmbH, Germany) containing 3 liters of the optimal medium and the temperature was maintained at 50°C. Fed-batch fermentations were performed in the 5-liter bioreactor containing 3 liters of the medium and a 30-liter bioreactor (Biotech-30BS, Shanghai Baoxing Inc. China) containing 20 liters of the medium. In the pulse feeding strategy, when the residual glucose concentration was below 20 g/liter, glucose powder (200 g) was added to the 5-liter bioreactor. Constant glucose-concentration feeding strategy was performed by pumping glucose solution (800 g/liter) into the bioreactor to maintain the residual glucose concentration at about 20 g/liter. Exponential feeding strategy was performed according that reported previously [19]. The feeding rate *F* was calculated based on the following values: *μ* = 0.51/h or 0.15/h, *V*_0_ = 3 liters, *Y*_x/s_ = 0.1, *S*_i_ = 800 g/liter. In all fed-batch fermentations, temperature was controlled at 50°C during the first 36 h and then shifted to 55°C gradually (0.2°C/h) to increase the solubility of calcium lactate and maintain the liquid state of the broth. Agitation was maintained at 200 rpm for batch and fed-batch fermentations. The pH was controlled at 5.6 by automatically adding CaCO_3_ slurry and no aeration was used in all batches. The preparation of pre-culture was the same as that in flask experiments and the inoculum volume was 10% (v/v). Fermentations were conducted open and without sterilization.

### Analytical methods

The concentrations of residual glucose and l-lactic acid were measured using an SBA-40C biosensor analyzer (Institute of Biology, Shandong Academy of Sciences, China). Dry cell weight was calculated from the optical density (OD_620 nm_) with a linear correlation factor (1 OD_620 nm_ = 0.565 g dry cell weight/liter). CaCO_3_ particles were eliminated by adding hydrochloric acid before measuring OD_620 nm_. High performance liquid chromatography (HPLC) analysis of l/d-lactic acid was performed on an Agilent 1100 series (Hewlett-Packard Corp., USA) equipped with a chiral column (MCI GEL CRS10W, Japan) and a tunable UV detector at 254 nm. The mobile phase was 2 mM CuSO_4_ at a flow rate of 0.5 ml/min (25°C). The optical purity of l-lactic acid was described as ee value which was defined as 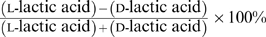. Byproducts were determined using HPLC equipped with a Bio-Rad Aminex HPX-87H column (300×7.8 mm) with a mobile phase of 6 mM H_2_SO_4_. The flow rate was 0.5 ml/min, and chromatographic peaks were detected in line at 205 nm. The column temperature was maintained at 55°C.

## Supporting Information

Table S1 Comparison of L-lactic acid optical purity produced by different bacteria
(0.03 MB DOC)

Supplementary Information S1 Statistical optimization of fermentation medium.
(0.14 MB DOC)

Figure S1 The response surface plot (A) and the corresponding contour plot (B) of L-lactic acid concentration (*Y*) as a function of soy peptide (*x*_1_) and YE (*x*_2_). ▴ represents the location where the maximized L-lactic acid concentration occurred; • represents raw data point.
(1.17 MB TIF)

Figure S2 The response surface plot (A) and the corresponding contour plot (B) of *Z* as a function of soy peptide (*X*_1_) and YE (*X*_2_) concentrations. ▴ represents the location where the maximized *Z* occurred.
(1.21 MB TIF)
