# Supplementary material for: Non-Sterilized Fermentative Production of Polymer-Grade L-Lactic Acid by a Newly Isolated Thermophilic Strain Bacillus sp. 2–6
Source: PLoS One. 2009 Feb 4;4(2):e4359. doi: 10.1371/journal.pone.0004359 (PMC2632756; doi:10.1371/journal.pone.0004359)
Supplement: Table S1 — Comparison of L-lactic acid optical purity produced by different bacteria (0.03 MB DOC) [file pone.0004359.s001.doc]

**Table S1.** Comparison of L-lactic acid optical purity produced by different bacteria

| Strains | D-Lactic acid (%) | L-Lactic acid (%) | Enantiomeric excess (%) |
| --- | --- | --- | --- |
| *Bacillus* sp. 2-6 | 1.35 | 98.65 | 97.30 |
| *Lactobacillus* *casei* DSM 20011 | 8.43 | 91.57 | 83.14 |
| *Lactobacillus* sp. DSM 20605 | 8.50 | 91.50 | 83.00 |
| *Lactobacillus* *plantarum* DSM 20205 | 44.84 | 55.16 | 10.32 |
| *Lactobacillus* *pentosus* DSM 20314 | 7.79 | 92.21 | 84.42 |
