# Supplementary material for: Non-Sterilized Fermentative Production of Polymer-Grade L-Lactic Acid by a Newly Isolated Thermophilic Strain Bacillus sp. 2–6
Source: PLoS One. 2009 Feb 4;4(2):e4359. doi: 10.1371/journal.pone.0004359 (PMC2632756; doi:10.1371/journal.pone.0004359)
Supplement: Supplementary Information S1 — Statistical optimization of fermentation medium. (0.14 MB DOC) [file pone.0004359.s002.doc]

# Supplementary information

**Statistical optimization of fermentation medium**

The optimization began with the selection of the right nitrogen sources. Soy peptide (Lenon Bio-Tech, Ltd, China), Yeast extract (YE) (Angel Yeast Co., Ltd, China) and cottonseed protein (Aoboxing Universeen Bio-Tech Co., Ltd, China ) were selected from six organic nitrogen sources as most suitable for *Bacillus* sp. 2-6 (data not shown). Effects of several inorganic nitrogen sources and trace elements on L-lactic acid production were investigated and the results showed that NaNO_3_, NH_4_Cl and Mg^2+^ had positive effect on L-lactic acid production when YE (5 g/liter) was used as the sole nitrogen source (Table 2). Many studies revealed that vitamins were required during lactic acid production by thermophilic *Bacillus* [15–17]. To study the effects of vitamins on L-lactic acid production by *Bacillus* sp. 2-6, vitamins were added separately to fermentation medium containing glucose (100 g/liter) and YE (5 g/liter). It was shown that when each of these vitamins was added, their effects on L-lactic acid production were not significant (data not shown). Therefore, a vitamin solution, along with soy peptide, YE, cottonseed protein and inorganic nitrogen sources, were used to compose the Plackett-Burman design (Tables 3 and 4).

The significance of each variable in the Plackett-Burman design was determined by *t* test (Table SI-1). Variables with confidence levels exceeding 90% (P < 0.10) were considered significant [18]. The analysis showed that soy peptide (*X*_1_) and YE (*X*_2_) had significant positive influence on L-lactic acid production, cottonseed protein (*X*_3_), NaNO_3_ (*X*_4_), NH_4_Cl (*X*_5_) had insignificant positive influence on L-lactic acid production, while Mg^2+^ (*X*_6_) and vitamin solution (*X*_7_) had negative influence on L-lactic acid production. A first-order model was obtained from the Plackett-Burman design experiments by least square fitting:

$Y=31.673+4.465x_{1}+9.160x_{2}+1.748x_{3}+0.827x_{4}+1.294x_{5}-0.373x_{6}-0.452x_{7}$ (1)

Analysis of variance (ANOVA) of the first-order model showed that the model used was suitable, with only 1.44% total variation that was not explained by the model (*R*^2^ = 0.9856). The *F* value and *P* value at 29.30 and 0.0092 also confirmed the significance of the model.

The optimal levels of the individual factors were determined by the following optimization steps. Variables with insignificant positive or negative effect were not included in the next optimization experiment, but used in all trials at their (+1) level and (–1) level, respectively. Then, the insignificant positive and negative terms in equation (1) were eliminated to give the following refined model:

$Y=31.673+4.465x_{1}+9.160x_{2}$ (2)

According to equation (2), the steepest ascent direction was proportional to (4.465, 9.160), approximately equivalent to (1, 2), meaning that if the soy peptide concentration increased one unit, the YE concentration would increase two units in order to approach the optimal region of both compositions at highest rate. In other words, the steepest ascent direction was a line passing through the center point (*x*_1_ = 0, *x*_2_ = 0) with a slope of 2. The L-lactic acid titer reached its maximum of 115.0 g/liter at *X*_1_ = 7 g/liter, *X*_2_ = 12 g/liter and this point would be an appropriate center for the second-order experiment (Table SI-2).

Central composite design was carried out around this point to optimize the levels of soy peptide and YE further to obtain the maximum L-lactic acid production (Table SI-3). Data obtained were analyzed by multiple regression and the experimental results of the central composite design were fitted and explained with a second-order polynomial function.

$Y=115-0.224x_{1}+8.510x_{2}-2.906x_{1}^{2}-6.625x_{1}x_{2}-15.406x_{2}^{2}$ (3)

The statistical significance of the equation was checked by *t* test (Table SI-4), and the results of ANOVA are shown in Table SI-5. The *F-*value of 77.29 and *P-*value of < 0.0001 imply that the model is a significant fit. The goodness of fit was expressed by the coefficient of determination *R*^2^, which was calculated to be 0.9822, indicating 98% of the variability in the response could be explained by the model. The value of Adj *R*^2^ (0.9695) was also very high to advocate for a high significance of the model. These results indicated that the response equation provided a suitable model for the central composite design.

The response surface plot (Figure S1A) and the corresponding contour plot (Figure S1B) described by the second-order model showed that the maximum value of L-lactic acid (116.7 g/liter) could be attained at soy peptide and YE concentrations of 5.1 g/liter (*x*_1_ = –0.4680) and 14.3 g/liter (*x*_2_ = –0.3768), respectively.

However, in our case the objective was not only to provide an efficient medium but also a cost-effective production medium. As shown in Table SI-3, 1.34 g/liter soy peptide and 12 g/liter YE resulted in 110.5 g/liter of L-lactic acid production, which is 94.8% of the maximum L-lactic acid concentration obtained at soy peptide and YE concentrations of 5.1 g/liter and 14.3 g/liter. But the former dosage of soy peptide and YE were only 26.3% and 83.9% of the latter. Therefore, it is necessary to balance the inconsistency between efficient and cost-effective.

To accomplish this objective, three preconditions were presented. Firstly, the difference between the cost of medium (only glucose, soy peptide and YE were considered) and the value of L-lactic acid produced was used as an indicator of the cost-effectiveness of the medium. Secondly, the final L-lactic acid concentration must be between 100.0 g/liter and 116.7 g/liter. In this range, the cost of downstream process was not affected by the insignificant change of L-lactic acid concentration. Thirdly, the weight conversion rate of glucose to L-lactic acid was 100%.

Based on these preconditions, a new variable *Z* describing the difference between L-lactic acid value and medium cost was presented and defined as:

*Z* = the value of product – the cost of medium

$Z=(Y\times w_{4}-Y\times w_{1}-X_{1}\times w_{2}-X_{2}\times w_{3})\times V\times10^{-3}$ (4)

where *Y* is the concentration of L-lactic acid or glucose (g/liter), *X*_1_, *X*_2_ are the concentrations of soy peptide and YE (g/liter), *w*_1_, *w*_2_, *w*_3_ and *w*_4_ are the price of glucose, soy peptide, YE and L-lactic acid (US$/kg), and *V* is the fermentation volume (liter).

Equation (5) is the uncoded form of equation (3). It can describe the relationship of L-lactic acid (*Y*), soy peptide (*X*_1_) and YE (*X*_2_) concentrations.

$Y=4.660+5.800X_{1}+13.621X_{2}-0.182X_{1}^{2}-0.276X_{1}X_{2}-0.428X_{2}^{2}$ (5)

Therefore, the combination of equation (4) and (5) resulted in:

$Z=V\times(w_{4}-w_{1})\left[4.66+(5.8-\frac{w_{2}}{w_{4}-w_{1}})\times X_{1}+(13.621-\frac{w_{3}}{w_{4}-w_{1}})\times X_{2}-0.182X_{1}^{2}-0.276X_{1}X_{2}-0.428X_{2}^{2}\right]\times10^{-3}$ (6)

In this research, the price of glucose (*w*_1_), soy peptide (*w*_2_), YE (*w*_3_) and L-lactic acid (*w*_4_) were 0.4 US$/kg, 3.0 US$/kg, 4.0 US$/kg and 2.0 US$/kg, respectively, according to the local market. Therefore, if the fermentation volume was 10^4^ liter, by solving the quadratic regression model equation, the maximum value of *Z* (1,233.8 US$) could be attained at soy peptide (*X*_1_) and YE (*X*_2_) concentrations of 1.2 g/liter and 12.6 g/liter, respectively. The response surface plot and the corresponding contour plot are shown in Figure S2. At top point, the concentration of L-lactic acid (*Y*) is 110.9 g/liter, amounting to 95.0% of the theoretical maximum L-lactic acid concentration. This medium will save 3.9 g/liter of soy peptide and 1.7 g/liter of YE and represents the most cost-effective medium.

According to the results of the statistically designed experiments and the investigation on initial glucose concentration, a cost-effective medium for L-lactic acid production by *Bacillus* sp. 2-6 was obtained: glucose 97-133 g/liter, YE 12.6 g/liter, soy peptide 1.2 g/liter, cottonseed protein 3 g/liter, NaNO_3_ 1 g/liter, NH_4_Cl 1 g/liter.

**Table SI-1.** Coefficients and *t* values calculated from the Plackett-Burman experiment

| Variable | Coefficient | Standard error | *t* value | *P* value |
| --- | --- | --- | --- | --- |
| Intercept | *β*_0_ = 31.6729 | 0.8083 | 39.18 | < 0.0001 |
| *x*_1_ | *β*_1_ = 4.4646 | 0.8083 | 5.52 | 0.0117 |
| *x*_2_ | *β*_2_ = 9.1604 | 0.8083 | 11.33 | 0.0015 |
| *x*_3_ | *β*_3_ = 1.7479 | 0.8083 | 2.16 | 0.1193 |
| *x*_4_ | *β*_4_ = 0.8271 | 0.8083 | 1.02 | 0.3815 |
| *x*_5_ | *β*_5_ = 1.2938 | 0.8083 | 1.60 | 0.2078 |
| *x*_6_ | *β*_6_ = –0.3729 | 0.8083 | –0.46 | 0.6759 |
| *x*_7_ | *β*_7_ = –0.4521 | 0.8083 | –0.56 | 0.6150 |

**Table SI-2.** Design and results of the steepest ascent experiment

| Step change value | Code levels ^a^ | | Real variables | | Response value |
| --- | --- | --- | --- | --- | --- |
|  | *x*_1_ | *x*_2_ | *X*_1_ ^b^  Soy peptide  (g/liter) | *X*_2_ ^c^  YE  (g/liter) | *Y*  L-Lactic acid  (g/liter) |
| Center point | 0 | 0 | 2 | 2 | 39.0 ± 0.7 ^d^ |
| Δ | 1 | 2 | 1 | 2 |  |
| Center point + Δ | 1 | 2 | 3 | 4 | 68.0 ± 1.4 |
| Center point + 2Δ | 2 | 4 | 4 | 6 | 87.0 ± 0.7 |
| Center point + 3Δ | 3 | 6 | 5 | 8 | 100.0 ± 2.1 |
| Center point + 4Δ | 4 | 8 | 6 | 10 | 112.0 ± 0.0 |
| Center point + 5Δ | 5 | 10 | 7 | 12 | 115.0 ± 0.7 |
| Center point + 6Δ | 6 | 12 | 8 | 14 | 112.0 ± 1.4 |
| Center point + 7Δ | 7 | 14 | 9 | 16 | 107.0 ± 1.4 |

^a^ Conveniently, the slope was changed to 2.

^b^ *X*_1_ is calculated as: *X*_1_ = 2 + *x*_1_ × 1.

^c^ *X*_2_ is calculated as: *X*_2_ = 2 + *x*_2_ × 1.

^d^ Values in Table SI-2 and Table SI-3 are the average ± standard deviation of three repeated fermentations.

**Table SI-3.** Design and results of the central composition experiment

| Code levels | | Real variables | | Response value |
| --- | --- | --- | --- | --- |
| *x*_1_ | *x*_2_ | *X*_1_ ^a^ | *X*_2_ ^b^ | *Y* |
|  |  | Soy peptide (g/liter) | YE  (g/liter) | L-Lactic acid  (g/liter) |
| –1 | –1 | 3 | 6 | 79.0 ± 1.4 |
| –1 | 1 | 3 | 18 | 112.5 ± 2.1 |
| 1 | –1 | 11 | 6 | 91.0 ± 1.4 |
| 1 | 1 | 11 | 18 | 98.0 ± 0.0 |
| –1.414 | 0 | 1.34 | 12 | 110.5 ± 0.7 |
| 1.414 | 0 | 12.66 | 12 | 111.0 ± 1.4 |
| 0 | –1.414 | 7 | 3.51 | 76.0 ± 2.8 |
| 0 | 1.414 | 7 | 20.49 | 95.5 ± 0.7 |
| 0 | 0 | 7 | 12 | 114.0 ± 1.4 |
| 0 | 0 | 7 | 12 | 115.0 ± 0.0 |
| 0 | 0 | 7 | 12 | 115.0 ± 1.4 |
| 0 | 0 | 7 | 12 | 116.0 ± 1.4 |
| 0 | 0 | 7 | 12 | 115.0 ± 0.0 |

^a^ *X*_1_ is calculated as: *X*_1_ = 7 + *x*_1_ × 4.

^b^ *X*_2_ is calculated as: *X*_2_ = 12 + *x*_2_ × 6.

**Table SI-4.** Coefficients and *t* values calculated from the central composition experiment

| Variable | Coefficient | Standard error | *t* value | *P* value |
| --- | --- | --- | --- | --- |
| Intercept | *β*_0_ = 115.0000 | 1.1173 | 102.93 | < 0.0001 |
| *x*_1_ | *β*_1_ = –0.2241 | 0.8833 | –0.25 | 0.8070 |
| *x*_2_ | *β*_2_ = 8.5096 | 0.8833 | 9.63 | < 0.0001 |
| *x*_1_^2^ | *β*_11_ = –2.9063 | 0.9472 | –3.07 | 0.0181 |
| *x*_1_*x*_2_ | *β*_12_ = –6.6250 | 1.2492 | –5.30 | 0.0011 |
| *x*_2_^2^ | *β*_22_ = –15.4062 | 0.9472 | –16.26 | < 0.0001 |

**Table SI-5.** ANOVA for evaluation of the second-order model

| Source of  variation | Degrees of  freedom | Sum of  squares | Mean  square | *F* value | *P* value |
| --- | --- | --- | --- | --- | --- |
| Model | 5 | 2412.1158 | 482.4232 | 77.29 | < 0.0001 |
| Residual | 7 | 43.6919 | 6.2417 |  |  |
| Total | 12 | 2455.8077 |  |  |  |

*R*^2^ = 0.9822, Adj *R*^2^ = 0.9695.
