# Supplementary figures and images for: Non-Sterilized Fermentative Production of Polymer-Grade L-Lactic Acid by a Newly Isolated Thermophilic Strain Bacillus sp. 2–6
Source: PLoS One. 2009 Feb 4;4(2):e4359. doi: 10.1371/journal.pone.0004359 (PMC2632756; doi:10.1371/journal.pone.0004359)

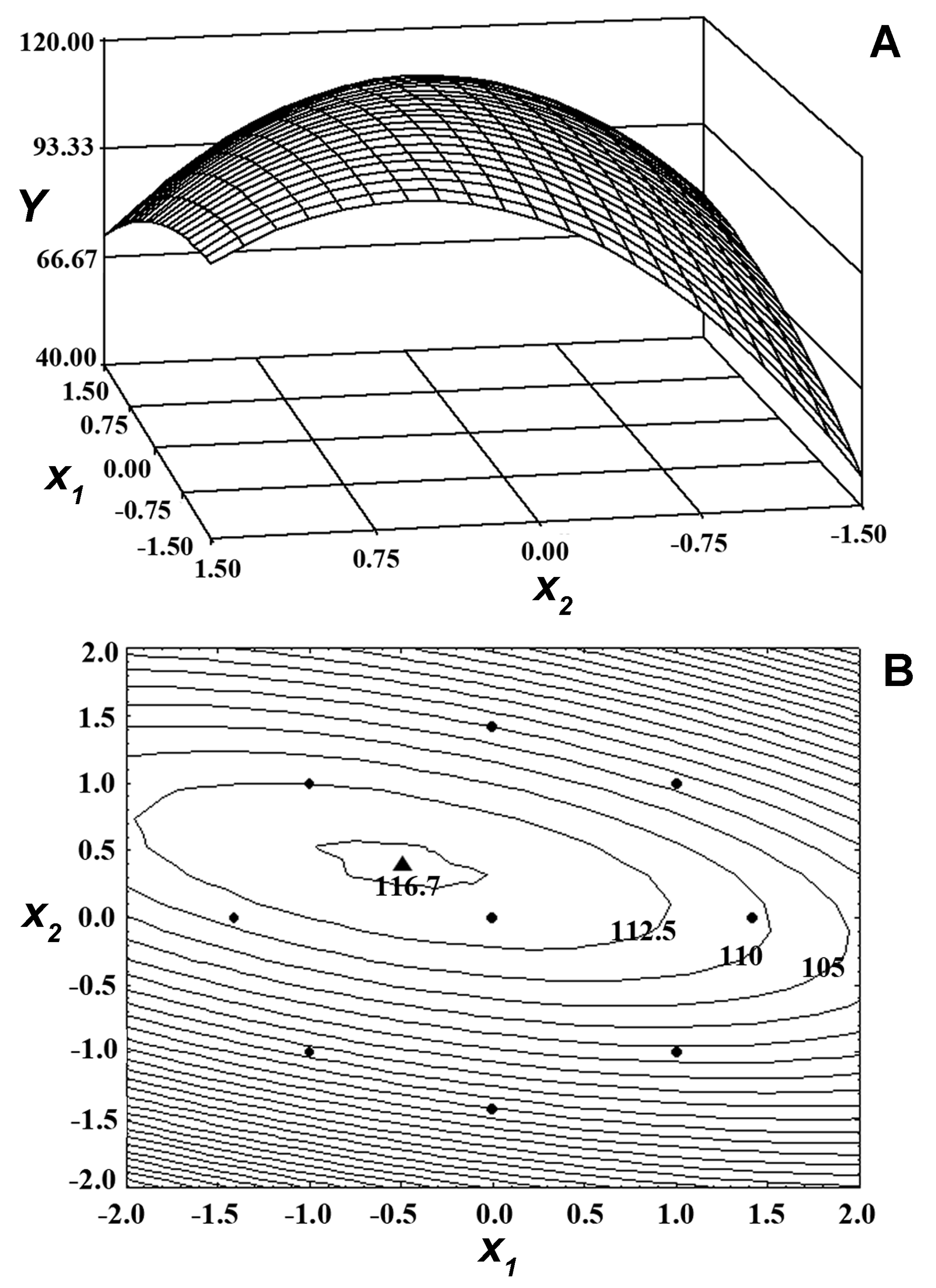

Supplement: Figure S1 — The response surface plot (A) and the corresponding contour plot (B) of L-lactic acid concentration (Y) as a function of soy peptide (x 1) and YE (x 2). ▴ represents the location where the maximized L-lactic acid concentration occurred; • represents raw data point. (1.17 MB TIF) [file pone.0004359.s003.tif]

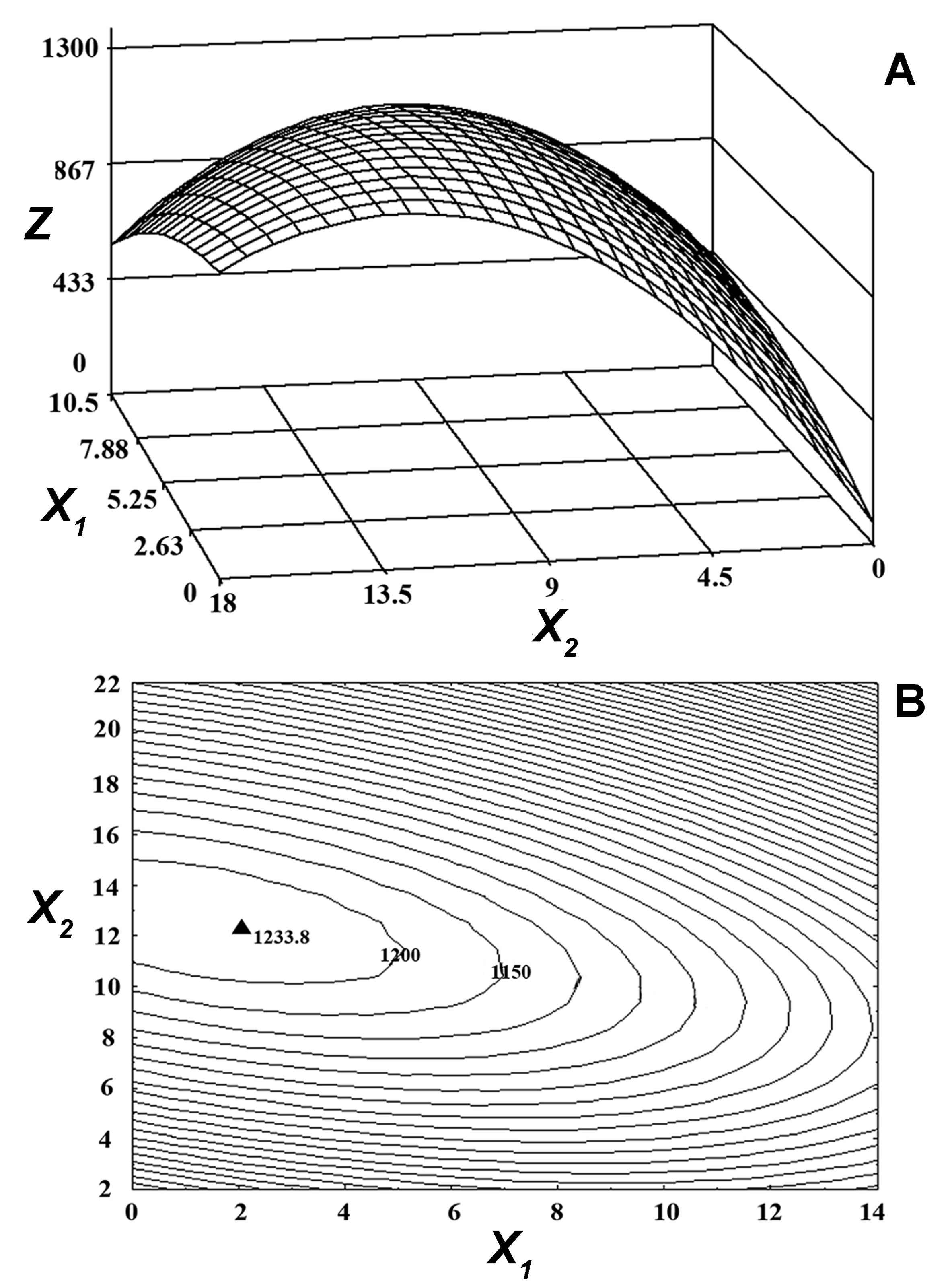

Supplement: Figure S2 — The response surface plot (A) and the corresponding contour plot (B) of Z as a function of soy peptide (X 1) and YE (X 2) concentrations. ▴ represents the location where the maximized Z occurred. (1.21 MB TIF) [file pone.0004359.s004.tif]
